# Acute nasal breathing lowers diastolic blood pressure and increases parasympathetic contributions to heart rate variability in young adults

**DOI:** 10.1152/ajpregu.00148.2023

**Published:** 2023-10-23

**Authors:** Joseph C. Watso, Jens N. Cuba, Savannah L. Boutwell, Justine E. Moss, Allison K. Bowerfind, Isabela M. Fernandez, Jessica M. Cassette, Allyson M. May, Katherine F. Kirk

**Affiliations:** Cardiovascular and Applied Physiology Laboratory, Department of Health, Nutrition, & Food Sciences, Florida State University, Tallahassee, Florida, United States

**Keywords:** blood pressure, blood pressure variability, breathing, cardiac vagal baroreflex, heart rate variability

## Abstract

There is growing interest in how breathing pace, pattern, and training (e.g., device-guided or -resisted breathing) affect cardiovascular health. It is unknown whether the route of breathing (nasal vs. oral) affects prognostic cardiovascular variables. Because nasal breathing can improve other physiological variables (e.g., airway dilation), we hypothesized that nasal compared with oral breathing would acutely lower blood pressure (BP) and improve heart rate variability (HRV) metrics. We tested 20 adults in this study [13 females/7 males; age: 18(1) years, median (IQR); body mass index: 23 ± 2 kg·m^−2^, means ± SD]. We compared variables between nasal- and oral-only breathing (random order, five min each) using paired, two-tailed *t* tests or Wilcoxon signed-rank paired tests with significance set to *P* < 0.05. We report the median (interquartile range) for diastolic BP and means ± SD for all other variables. We found that nasal breathing was associated with a lower mean BP (nasal: 84 ± 7 vs. oral: 86 ± 5 mmHg, *P* = 0.006, Cohen’s *d* = 0.70) and diastolic BP [nasal: 68(8) vs. oral: 72(5) mmHg, *P* < 0.001, Rank-biserial correlation = 0.89] but not systolic BP (nasal: 116 ± 11 vs. oral: 117 ± 9 mmHg, *P* = 0.48, Cohen’s *d* = 0.16) or heart rate (HR; nasal: 74 ± 10 vs. oral: 75 ± 8 beats·min^−1^, *P* = 0.90, Cohen’s *d* = 0.03). We also found that nasal breathing was associated with a higher high-frequency (HF) contribution to HRV (nasal: 59 ± 19 vs. oral: 52 ± 21%, *P* = 0.04, Cohen’s *d* = 0.50) and a lower low frequency-to-HF ratio at rest (nasal: 0.9 ± 0.8 vs. oral: 1.2 ± 0.9, *P* = 0.04, Cohen’s *d* = 0.49). These data suggest that nasal compared with oral breathing acutely *1*) lowers mean and diastolic BP, *2*) does not affect systolic BP or heart rate, and *3*) increases parasympathetic contributions to HRV.

**NEW & NOTEWORTHY** There is growing interest in how breathing pace, pattern, and training (e.g., device-guided or -resisted breathing) affect prognostic cardiovascular variables. However, the potential effects of the breathing route on prognostic cardiovascular variables are unclear. These data suggest that nasal compared with oral breathing *1*) lowers mean and diastolic blood pressure (BP), *2*) does not affect systolic BP or heart rate (HR), and *3*) increases parasympathetic contributions to heart rate variability (HRV). These data suggest that acute nasal breathing improves several prognostic cardiovascular variables.

## Graphical abstract

**Figure:**
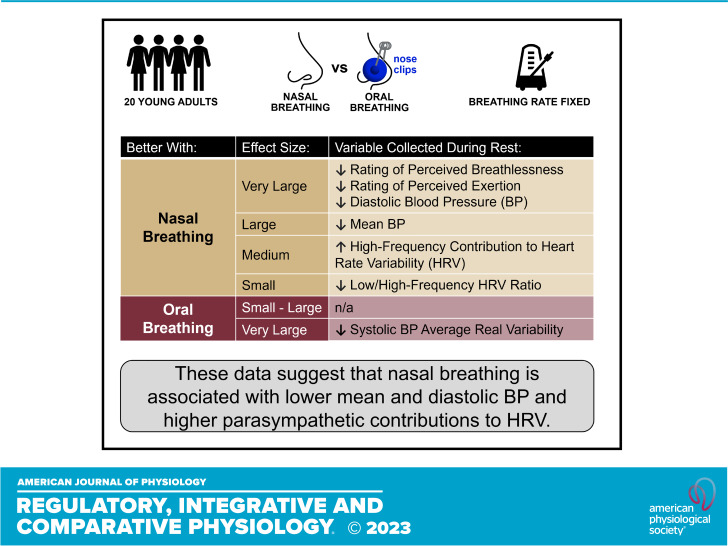

## INTRODUCTION

The leading cause of death in the United States is cardiovascular disease (1). Cardiovascular disease risk can be predicted by blood pressure (BP) (1), heart rate variability (HRV) (2, 3), blood pressure variability (BPV) (4–11), and cardiac vagal baroreflex sensitivity (cBRS) (12–14). These prognostic cardiovascular variables can be affected by respiration because of the dynamic interplay between the cardiovascular and respiratory systems. Broadly, these systems make internal adjustments to match alveolar ventilation with cardiac output (15).

Cardiac output is the product of stroke volume and heart rate (HR). HR is carefully regulated by the autonomic nervous system via changing the R-R interval (i.e., length of the cardiac cycle) (16). Such variation in the R-R interval is quantified as HRV. HRV is commonly examined during paced breathing (17–24) because the rate of breathing can affect HRV metrics in the time [e.g., respiratory sinus arrhythmia (25)] and frequency (high-frequency component) domains (24, 26–31). R-R interval variation is partly dependent on the arterial baroreflex. The arterial baroreflex control of HR is quantified as the slope between changes in the R-R interval in response to changes in systolic BP (i.e., cBRS). Similar to HRV, past research has used paced breathing to examine cBRS (17, 22, 23) because slow breathing raises (i.e., improves) cBRS (32, 33). Likewise, recent work has used paced breathing to study BP and BPV (5, 32–34). Although many have examined how breathing rate (5, 19–23, 26, 28–30, 32–34) affects BP, HRV, BPV, and cBRS, it is unclear whether the breathing route (nasal vs. oral) affects these prognostic cardiovascular variables.

Inhaled air is humidified, warmed, and filtered with nasal breathing (35). Nasal breathing can elicit bronchodilation (36), potentially due to greater airway epithelium nitric oxide production (37). Nasal breathing also increases diaphragmatic movement and reduces the recruitment of accessory inspiratory muscles (38), which may explain lower resting metabolic demands (i.e., V̇o_2_, rate of oxygen uptake) reported during nasal versus oral breathing (39). Although nasal breathing relaxes the airways, improves breathing efficiency, and reduces metabolic demands, the effect of nasal breathing on the cardiovascular system is unclear. One of the aforementioned studies (39) did not find nasal breathing to affect resting HR. However, other important prognostic markers of cardiovascular health may be improved during acute nasal versus oral breathing. Therefore, we sought to test the primary hypothesis that nasal, compared with oral, breathing would decrease BP, improve HRV metrics, reduce BPV, and increase cBRS in young adults. This is a timely question to complement the growing interest in how breathing pace, pattern, and training (e.g., device-guided or -resisted breathing) affect prognostic cardiovascular variables (40–46).

If nasal breathing improves cardiovascular variables at rest, these variables could also improve when the exercise pressor reflex is active. For example, published work demonstrates a strong relation between lower (impaired) cBRS values and higher (unfavorable) BP responses to phenylephrine, another BP-raising stimulus (47). Past research reported no difference in HR between nasal and oral breathing during submaximal exercise (48, 49). However, it remains unclear whether nasal breathing affects BP or components of BP regulation (e.g., HRV, BPV, cBRS) during submaximal exercise. This has clinical relevance because higher exercise BP values are associated with a greater risk for the future development of hypertension and cardiovascular disease (50–53). Because past work suggests that nasal breathing attenuates the ventilatory response to exercise (39, 49, 54) and metabolic demands (39, 49), our secondary hypothesis was that nasal compared with oral breathing would attenuate BP responses, improve HRV metrics, and reduce BPV during exercise.

## METHODS

### Ethical Approval

The Institutional Review Board for Human Subjects Research at Florida State University approved this protocol and the associated informed consent (IRB No. 3661). This study conformed with the standards set by the latest revision of the Declaration of Helsinki. Each of the 20 participants provided verbal and written consent before enrollment in the study. The data in this manuscript are associated with a registered clinical trial (ClinicalTrials.gov Identifier: NCT05702047).

### Participants

We recruited participants from Tallahassee, FL. All participants who enrolled in this study were free of any known cardiovascular, metabolic, or neurological diseases. Inclusion criteria included age between 18 and 30 yr old, seated blood pressure ≤140/90 mmHg, and body mass index <30 kg·m^−2^. Exclusion criteria included the presence of overt cardiovascular (e.g., diagnosed hypertension), respiratory, neurological, renal, liver, and/or metabolic health conditions. Current or recent (regular use within the past 6 mo) users of tobacco or nicotine products (e.g., cigarettes) were also excluded. We used a Gulick tape measure to assess waist and hip circumferences as an index of body composition. Finally, we used the International Physical Activity Questionnaire (IPAQ)-Short Form as an index of habitual physical activity in 18 participants (55).

### Experimental Protocol

**Figure 1.**
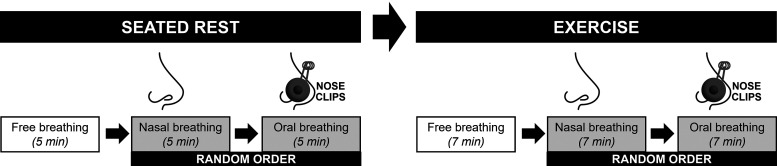
Experimental design. Participants visited the laboratory on one occasion for consent, screening, and testing. The experimental protocol included three 5-min periods at rest with free breathing (to measure participants’ self-selected breathing rate) before nasal-only and oral-only breathing in random order. Next, participants completed the same paradigm (free breathing then randomized nasal and oral breathing) during three 7-min periods while cycling at 75 W on a lower-body ergometer.

This study was designed as a randomized, crossover, counterbalanced trial with one experimental visit including informed consent, screening, assessment of cardiovascular variables during nasal and oral breathing, and then a peak exercise test (Fig. 1). Laboratory conditions were 22.0 ± 1.2°C for ambient temperature, 52 ± 14% for relative humidity, and 765 ± 3 mmHg for atmospheric pressure. We instructed participants to avoid food for at least 2 h before the trial as well as caffeine, alcohol, and strenuous exercise 24 h before each trial. Any supplements, medications, or vitamins taken by participants were not withheld before the testing visit. We did not select one specific menstrual cycle in female participants to complete testing. Participants were instrumented with equipment to measure heart rate and blood pressure. We used a strain-gauge pneumograph to measure respiratory excursions at the chest and determine the respiratory rate (Respiratory Belt Transducer, ADInstruments, Colorado Springs, CO). We also used a standard finger clip to estimate blood oxygen saturation (Oximeter Pod, ADInstruments, Colorado Springs, CO) in 19 participants at rest as one was excluded due to technical difficulties. Finally, we oriented participants to verbally report their rating of perceived exertion (RPE) with values of 6 “no exertion” to 20 “maximal exertion” (56), and their rating of perceived breathlessness (RPB) with values of 0 “nothing at all” to 10 “maximal” (57) displayed as a standard vertical list of numerical values and descriptors (e.g., 0, nothing at all).

### Phase 1: Resting Protocol

Participants relaxed quietly in a semirecumbent position for 5 min with no breathing cues. Next, we set an auditory metronome to twice an individual’s self-selected respiratory rate (e.g., 12 breaths·min^−1^ = 24 beats·min^−1^). We instructed participants to begin an inspiration or expiration with each beat (i.e., 50% time inhaling and 50% time exhaling) to fix their respiratory rate for the two conditions of interest completed in random order. One condition included nasal-only breathing for 5 min with participants instructed to only breathe through their nose and to keep their lips sealed, for which compliance was confirmed by simply watching the participant breathe. The other condition included oral-only breathing for 5 min with participants fitted with a soft nose clip that prevented nasal airflow. We measured brachial BP, RPE, and RPB during the final minute of each 5 min.

### Phase 2: Submaximal Exercise Protocol

Participants cycled at 75 W for 7 min on an Ergoline 600 K Recumbent Lower-body Cycle Ergometer (COSMED USA, Inc., Concord, CA) with no breathing cues. Next, we set an auditory metronome to twice an individual’s self-selected respiratory rate (e.g., 24 breaths·min^−1^ = 48 beats·min^−1^). We instructed participants to begin an inspiration or expiration with each beat to fix their respiratory rate for the two conditions of interest completed in random order. One condition included nasal-only breathing for 7 min with participants instructed to only breathe through their nose and to keep their lips sealed, for which compliance was confirmed by simply watching the participant breathe. The other condition included oral-only breathing for 7 min with participants fitted with a soft nose clip that prevented nasal airflow. We measured brachial BP during the 5th and 6th minute of exercise, with the two values being averaged for analysis. Finally, we measured RPE and RPB during the final minute of each 7 min.

We selected 75 W [∼5.5 metabolic equivalents (METs)] to mimic moderate-intensity exercise with 6 METs being considered the accepted cutoff for vigorous-intensity exercise (58). This fixed-workload approach has ecological validity to activities of daily living, with 75 W on a cycle being comparable in metabolic demands to walking at 3.2 mph at a 1% grade. In addition, 75 W is below the typical oronasal breathing switch point that occurs at workloads above 100 W (59, 60) regardless of biological sex (59, 61). Finally, we selected 7-min stages based on past work (62–64) suggesting that completing 6 min of steady-state exercise is valid for RPB values.

### Phase 3: Peak Exercise Protocol

After submaximal exercise testing, participants took a break for a self-selected duration (e.g., 5–10 min) before the peak exercise test. We instructed participants to breathe freely with no specific route (nasal vs. oral) or pacing. Starting at 50 W, the cycling workload increased by 25 W·min^−1^ until test termination. The test was terminated when participants could no longer continue or maintain 60 rpm despite strong verbal encouragement. We report the wattage of the final completed stage as an index of their peak work capacity. To provide context with the peak heart rate data, we estimated the maximal heart rate as 220 minus age in years to calculate the percent of heart rate maximum achieved.

### Brachial Blood Pressure and HRV

We used an automated BP monitor (HEM-907XL, OMRON, Kyoto, Japan) to measure brachial BP in the right arm of all participants at rest. We excluded three participants from the analysis during submaximal exercise because of invalid readings from arm movement in two participants and discontinued the exercise protocol after lightheadedness was reported by one participant. As previously described (65–67), we used single-lead ECG to continuously assess heart rate and heart rate variability (BioAmp, ADInstruments, Colorado Springs, CO). We calculated the rate pressure product during exercise as systolic BP multiplied by heart rate during exercise. Data were collected at a sampling rate of 1,000 Hz using LabChart Pro (ADInstruments, Colorado Springs, CO) (68) and analyzed using the Kubios HRV software (v.3.5). We set automatic noise detection to “medium” within the program. Our analysis included time and frequency domain HRV measures. Our time-domain measures included the standard deviation of NN intervals (SDNN), the percentage of detected NN intervals greater than 50 ms different from the immediately preceding NN interval (pNN50), and the root mean square of successive differences (RMSSD). Our frequency domain measures included low-frequency absolute power (LF; ms^2^) and relative contribution to total power (%), high-frequency absolute power (HF; ms^2^) and relative contribution to total power (%), and the LF/HF contribution ratio. We defined the range of each frequency band as LF range 0.04–0.15 Hz and HF range 0.15–0.40 Hz (69, 70). Because of the technical difficulties of keeping clear ECG readings during exercise (i.e., increased movement and reduced adherence of electrodes to the skin, etc.), we excluded one participant from our exercise HRV analysis.

### Beat-to-Beat Hemodynamic Measures

We used finger photoplethysmography to assess beat-to-beat BP and Modelflow-derived cardiac output (Finometer NOVA; Finapres Medical Systems, The Netherlands) (71), as previously described (72–79), in 13 participants after equipment failure in the initial seven visits. Briefly, Modelflow is a three-element model that uses arterial characteristic impedance, arterial compliance, and peripheral resistance to compute valid estimates of stroke volume (80). Thus, Modelflow allows for valid estimates of cardiac output (81). We defined BP waveform peaks as systolic BP, nadirs as diastolic BP, and the average value of the integrated BP waveform as mean BP. Total vascular conductance was calculated by dividing cardiac output by finometer-derived mean BP.

### Blood Pressure Variability

We calculated the standard deviation of BP from the subset of participants with beat-to-beat hemodynamic data at rest because BPV is prognostic for cardiovascular morbidities (4–11), inclusive of during exercise BPV (82). We also calculated the average real variability index, which is the average of absolute differences between consecutive BP measurements. This index provides further prognostic value compared with traditional measures of BPV, such as the standard deviation of BP (83, 84). We also calculated BPV in 11 participants with satisfactory beat-to-beat BP data who completed submaximal exercise testing.

### Resting Cardiac Vagal Baroreflex Sensitivity

As previously described (65, 66, 73), we also analyzed beat-to-beat time series of systolic BP and R-R intervals using the sequence method for estimating spontaneous cardiac vagal baroreflex gain (HemoLab v.23.8; Harald Stauss Scientific, Iowa City, IA). A detailed description of this method has been published previously (85). Briefly, we identified sequences of four or more consecutive cardiac cycles in which systolic BP and R-R interval change in the same direction. Sequences were detected only when the variation in the R-R interval was >0.5 ms and systolic BP changes were >1 mmHg. We applied a linear regression to each sequence, and only those sequences in which *i*^2^ was >0.80 were accepted. We averaged the slopes of those individual linear regressions for both up (increase in both systolic BP and R-R interval) and down (decrease in both systolic BP and R-R interval) sequences. We used data from 10 participants who had beat-to-beat BP data and ≥3 up sequences and 11 participants with ≥3 down sequences during the collection periods. We calculated overall spontaneous cardiac vagal baroreflex sensitivity by averaging all sequences for each of ten individuals with ≥3 up sequences and ≥3 down sequences.

### Data and Statistical Analysis

We analyzed all 5 min of the 5-min rest periods. There was no a priori power analysis for this investigation. To address our primary hypothesis, we compared variables collected during rest using paired, two-tailed *t* tests or Wilcoxon matched-pairs signed-rank tests when data failed (*P* > 0.05) the Shapiro–Wilk test for normality. We calculated the proportion of adults reporting an RPE over 6 between conditions at rest using a two-sided Fisher’s exact test. We correlated changes (nasal minus oral breathing values) in diastolic BP and other variables that changed between conditions (e.g., change in diastolic BP vs. change in LF/HF ratio). To address our secondary hypothesis, we compared variables collected during exercise using paired, two-tailed *t* tests or Wilcoxon matched-pairs signed-rank tests when data failed (*P* > 0.05) the Shapiro–Wilk test for normality. We analyzed the final 5 min of the 7-min exercise periods to avoid the initial transient response at the start of the exercise.

We calculated effect sizes to aid with interpretation (86, 87) in addition to the calculated *P* values. We interpreted Cohen’s *d* effect sizes as small (0.20–0.49), medium (0.50–0.79), and large (>0.80) (88) for normally distributed data and interpreted the Rank-biserial correlation (*r*_bc_) effect sizes as small (0–0.19), medium (0.20–0.29), large (0.30–0.39), and very large (0.40–1) (89) for non-normally distributed data. We present values as means ± SD or median (interquartile range) for data that are not normally distributed. We analyzed data using Jamovi [an internationally developed open-source project (90)] and GraphPad Prism (v.9.4.0 for Windows, GraphPad Software, San Diego, CA).

### Exploratory Analysis

On an exploratory basis (see *Predicting diastolic BP reductions with nasal breathing from resting data*), we also performed linear regression to determine potential predictors of the observed nasal breathing-induced diastolic BP lowering with the following variables: peak cycling workload, waist-to-hip ratio (WHR), screening diastolic BP, and biological sex. We removed variables [in order of highest variance inflation factor (VIF)] until all variables in the model were ≤1.20 to remove the confounding effects of multicollinearity.

## RESULTS

### Participants

We present participant screening characteristics in Table 1. We present the participant’s self-reported physical activity and peak cycling test results in Table 2.

**Table 1.** Participant screening information

Number of participants	20
Biological sex	13 females, 7 males
Race	1 Black, 19 White
Ethnicity	3 Hispanic, 17 non-Hispanic
Age, yr	18 [1]
Body mass, kg	65 ± 10
Body mass index, kg·m^−2^	23 ± 2
Waist-to-hip ratio	0.77 [0.07]
Brachial systolic BP, mmHg	116 ± 12
Brachial diastolic BP, mmHg	67 ± 7

We present data as median [interquartile range] or means ± SD.

**Table 2.** Physical activity habits and peak exercise test data

Moderate-to-vigorous physical activity, min·wk^−1^	320 [600]
Workload, W	186 ± 50
Heart rate, beats·min^−1^	184 ± 8
Heart rate, % of maximum heart rate	91 ± 4
Rating of perceived exertion	17 ± 2
Rating of perceived breathlessness	6 ± 2

We present data as median [interquartile range] or means ± SD.

### Resting Respiratory Rate, Brachial Blood Pressure, and Hemodynamics

**Figure 2.**
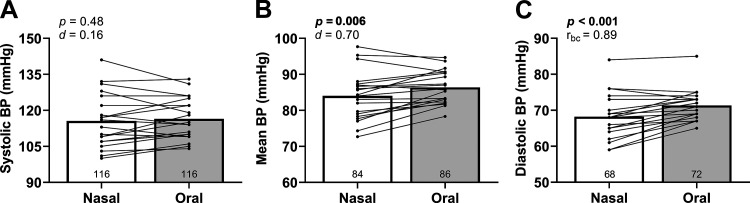
Brachial blood pressure during rest. *A*: systolic blood pressure (BP) was not different between conditions. Mean (*B*) and diastolic (*C*) BP were lower during nasal breathing. We used two-tailed, paired *t* tests for all tests except diastolic BP (Wilcoxon test). *n* = 20 for all graphs.

Respiratory rate was not different between conditions (nasal: 15 ± 4 vs. oral: 15 ± 4 breaths·min^−1^, *P* = 0.08, *d* = 0.42). Systolic BP was not different between conditions (Fig. 2*A*). However, mean BP and diastolic BP were lower with nasal breathing (Fig. 2, *B* and *C*). Model flow-derived estimates of cardiac output (nasal: 8.8 ± 3.4 vs. oral: 8.9 ± 3.5 L·min^−1^, *P* = 0.36, *d* = 0.26) and total vascular conductance (nasal: 0.10 ± 0.04 vs. oral: 0.10 ± 0.04 L·min^−1^·mmHg^−1^, *P* = 0.64, *d* = 0.14) were not different between conditions. Oxygen saturation was also not different between conditions [nasal: 97.9 (1.4) vs. oral: 97.8 (1.2)%, *P* = 0.74, *r*_bc_ = 0.09].

### Resting Heart Rate and HRV

**Figure 3.**
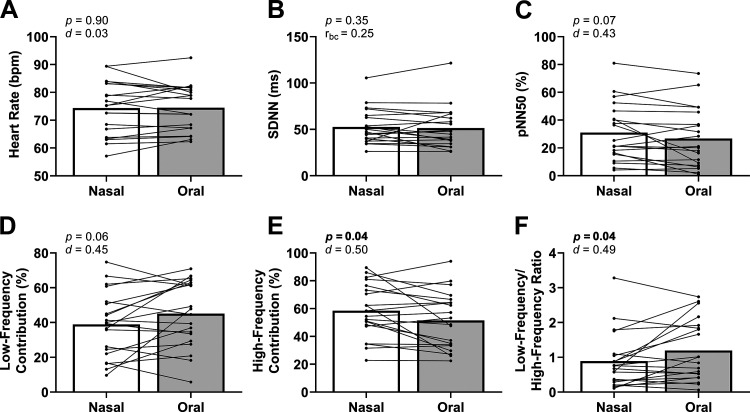
Heart rate variability metrics during rest. Heart rate (*A*), the standard deviation of NN intervals (SDNN, *B*), the percentage of detected NN intervals greater than 50 ms different from the immediately preceding NN interval (pNN50, *C*), and the low-frequency (LF) contribution were not different between conditions (*D*). The high-frequency (HF) contribution was lower (*E*) and the low-frequency (LF)/HF ratio (*F*) was higher during nasal breathing. We used two-tailed, paired *t* tests for all tests except SDNN (Wilcoxon test). *n* = 20 for all graphs.

Heart rate, SDNN, pNN50, and LF contribution were not different between conditions (Fig. 3, *A*–*D*). HF contribution was higher and LF/HF was lower during nasal breathing (Fig. 3, *E* and *F*). RMSSD, LF power, and HF power were not different between conditions (Table 3).

**Table 3.** Heart rate variability, blood pressure variability, and subjective ratings during rest

	Nasal	Oral	*P*	Effect Size
Secondary heart rate variability metrics (*n* = 20)
RMSSD, ms	46 [28]	42 [31]	0.15	*r*_bc_ = 0.37
Low-frequency power, ms^2^	697 [1,214]	858 [544]	0.93	*r*_bc_ = 0.03
High-frequency power, ms^2^	1,347 [1,371]	1,082 [1,771]	0.90	*r*_bc_ = 0.04
BP variability (*n* = 13)
Systolic BP average real variability, mmHg	2.7 [2.2]	2.5 [1.4]	**0.05**	*r*_bc_ = 0.63
Mean BP average real variability, mmHg	1.7 [0.8]	1.5 [1.8]	0.13	*r*_bc_ = 0.50
Diastolic BP average real variability, mmHg	1.7 ± 0.4	1.6 ± 0.4	0.26	*d* = 0.33
Systolic BP standard deviation, mmHg	6.1 [2.3]	6.1 [2.4]	0.74	*r*_bc_ = 0.12
Mean BP standard deviation, mmHg	4.8 ± 0.7	4.7 ± 1.2	0.76	*d* = 0.09
Diastolic BP standard deviation, mmHg	4.4 ± 0.8	4.4 ± 1.6	>0.99	*d* < 0.01
Subjective ratings (*n* = 20)				
Rating of perceived exertion	6 [0]	6 [1]	**0.03**	*r*_bc_ = 1.00
Rating of perceived breathlessness	0.0 [0.0]	0.5 [0.5]	**<0.01**	*r*_bc_ = 1.00

We present data as median [interquartile range] or means ± SD. BP, blood pressure; RMSSD, root mean square of successive differences. *d*, Cohen’s *d*; *r*_bc_, rank-biserial correlation.

### Resting BPV and cBRS

Systolic, but not mean or diastolic BP, average real variability was higher during nasal breathing (Table 3). Systolic, mean, and diastolic BP standard deviations were not different between conditions (Table 3). The sensitivity (i.e., gain) of cardiac vagal baroreflex up (nasal: 15 ± 7 vs. oral: 15 ± 7 ms·mmHg^−1^, *P* = 0.87, *d* = 0.06), down (nasal: 14 ± 6 vs. oral: 14 ± 6 ms·mmHg^−1^, *P* = 0.93, *d* = 0.03), and all (nasal: 14 ± 6 vs. oral: 15 ± 7 ms·mmHg^−1^, *P* = 0.90, *d* = 0.04) sequences were not different between conditions.

### Resting RPE and RPB

RPE and RPB were lower during nasal breathing (Table 3). A greater proportion of adults reported an RPE above 6 (i.e., 7 or 8) during oral compared with nasal breathing (*P* = 0.04).

### Resting Diastolic BP Correlations

We did not find any meaningful correlations between diastolic BP changes and HF contribution, LF/HF ratio, RPE, and RPB changes between conditions (*r*s ≤ 0.09, *P*s ≥ 0.27 for all four correlations). Related, the reduction in diastolic BP from oral to nasal breathing did not differ (*P* = 0.44, data not shown) between those who were randomized to oral breathing first and those who were randomized to nasal breathing first.

### Exercise Data

Respiratory rate was not different between conditions (nasal: 25 ± 4 vs. oral: 24 ± 4 breaths·min^−1^, *P* = 0.09, *d* = 0.40). Systolic, mean, and diastolic BP were not different between conditions (Table 4). Modelflow-derived estimates of cardiac output (nasal: 15.0 ± 5.6 vs. oral: 15.6 ± 5.1 L·min^−1^, *P* = 0.09, *d* = 0.57) and total vascular conductance (nasal: 0.15 ± 0.06 vs. oral: 0.15 ± 0.05 L·min^−1^·mmHg^−1^, *P* = 0.76, *d* = 0.09) were not different between conditions. Heart rate, SDNN, pNN50, LF contribution, HF contribution, and LF/HF were not different between conditions (Table 4). RMSSD, LF power, HF power, and pNN50 (data not shown, *P*s ≥ 0.31) and indices of beat-to-beat BP variability (data not shown, *P*s ≥ 0.11) were not different between conditions. The rate pressure product did not differ between conditions (nasal: 18,692 ± 3,998 vs. oral: 19,789 ± 4,553 mmHg·beats·min^−1^, *P* = 0.31, *d* = 0.26). RPB, but not RPE, was lower during nasal breathing (Table 4). Oxygen saturation was not different between conditions (nasal: 96.3 ± 1.9 vs. oral: 96.7 ± 2.1%, *P* = 0.08, *d* = 0.43).

**Table 4.** Blood pressure, key heart rate variability metrics, and subjective ratings during exercise

	Nasal	Oral	*P*	Effect Size
Brachial blood pressure (*n* = 17)
Systolic BP, mmHg	142 ± 14	146 ± 12	0.27	*d* = 0.28
Mean BP, mmHg	98 ± 11	101 ± 12	0.35	*d* = 0.23
Diastolic BP, mmHg	77 ± 13	78 ± 13	0.65	*d* = 0.11
Key heart rate variability metrics (*n* = 18)
Heart rate, beats·min^−1^	130 ± 20	133 ± 22	0.45	*d* = 0.18
SDNN, ms	7.4 [6.9]	6.5 [8.4]	0.47	*r*_bc_ = 0.21
Low-frequency contribution, %	66 ± 12	69 ± 12	0.17	*d* = 0.34
High-frequency contribution, %	21 ± 14	19 ± 11	0.45	*d* = 0.18
Low-frequency/High-frequency ratio	3.5 [3.3]	4.0 [6.7]	0.64	*r*_bc_ = 0.11
Subjective ratings (*n* = 18)
Rating of perceived exertion	11 ± 3	12 ± 2	0.10	*d* = 0.42
Rating of perceived breathlessness	2.0 [1.5]	3.0 [2.3]	**0.03**	*r*_bc_ = 0.74

We present data as median [interquartile range] or means ± SD. BP, blood pressure; SDNN, standard deviation of NN intervals. *d*, Cohen’s *d*; *r*_bc_, rank-biserial correlation.

### Exploratory Analysis

#### Predicting diastolic BP reductions with nasal breathing from resting data.

The initial model for predicting nasal breathing-induced diastolic BP lowering resulted in *R*^2^ = 0.53, adjusted *R*^2^ = 0.39, *P* = 0.02 with peak cycling workload, WHR, screening diastolic BP, and biological sex. We removed biological sex (variance inflation factor of 2.93), resulting in *R*^2^ = 0.52, adjusted *R*^2^ = 0.42, *P* = 0.01 with peak cycling workload, WHR, and screening diastolic BP. For model coefficients, the intercept (estimate: −36.2, *P* = 0.008) and peak cycling workload (estimate: −0.03, *P* = 0.04) were negative predictors, WHR was a positive predictor (estimate: 29.8, *P* = 0.01), and screening diastolic BP was a nonsignificant positive predictor (estimate: 0.2, *P* = 0.15).

## DISCUSSION

Our primary novel finding was that nasal compared with oral breathing improved several physiological and subjective variables at rest and during exercise. At rest, nasal breathing was associated with lower mean BP, diastolic BP, LF/HF ratio, RPE, and RPB as well as a higher HF contribution to HRV. Conversely, nasal breathing increased (i.e., worsened) systolic BP average real variability. During submaximal exercise, there was a lack of a difference between conditions for nearly all variables assessed except for nasal breathing being associated with lower RPB. We interpret the collective data to suggest that nasal compared with oral breathing provides modest, but potentially clinically relevant, improvements in prognostic cardiovascular variables at rest, but not during exercise.

### Resting Blood Pressure

We found that nasal breathing was associated with a lower mean (large effect size) and diastolic (very large effect size) BP but did not affect systolic BP. We interpret the acute median reduction in diastolic BP of 4 mmHg as a modest benefit of potential clinical importance. As for the mechanisms leading to the reduction in BP observed with nasal breathing, we posited that this could be associated with the observed increases in HF contribution to HRV because previous work has linked a higher HF contribution to HRV with lower diastolic BP (91, 92). However, we did not find any significant correlations between changes in BP and any HRV metric (see *Resting Heart Rate and HRV*). Of note, one additional variable not assessed in the present study due to methodological considerations is metabolic demand (e.g., rate of oxygen uptake or V̇o_2_). This is relevant because another study reported that participants, particularly males, had a lower V̇o_2_ (without a difference in minute ventilation) during nasal breathing at rest (35). Nasal breathing induces bronchodilation (32) via airway epithelium nitric oxide production (33) and increases diaphragmatic movement (34). Greater diaphragmatic involvement and bronchodilation may be required to increase the pressure gradient and maintain airflow as nasal resistance is twice that of the lower airway during nasal breathing (93). This speculation should be confirmed in prospective mechanistic studies.

Although we did not assess these variables in this initial investigation, we did observe small, yet significant, reductions in ratings of exertion and breathlessness during nasal versus oral breathing at rest. With the present results in mind, future work can address any potential connections between these respiratory variables and BP changes by simultaneously measuring additional variables.

Other acute breathing interventions have reported comparable changes in BP. For example, acute slow breathing (e.g., 6–8 breaths·min^−1^) lowers diastolic BP by 1–5 mmHg on average (33, 42, 94–97). Furthermore, chronic interventions also reduce diastolic BP. For example, 10–15 min daily of musically guided breathing for 8 wk lowered diastolic BP by 2–7 mmHg (98–101). Indeed, a recent review detailed that device-guided slow breathing is a feasible and effective approach to lower BP (41). For device-resisted breathing studies, there is emerging literature supporting inspiratory muscle strength training (slowed breathing with resistance during inhalations) to lower diastolic BP by ∼4 mmHg (102). Together, several approaches to modulating breathing seem to reduce diastolic BP acutely and chronically.

This foundational data set in young adults can be extended to inform studies to examine whether other groups (e.g., adults with obesity, older adults, etc.) exhibit BP reductions. Furthermore, these data support future work to determine whether chronic nasal breathing is beneficial for BP (e.g., several acute bouts throughout the day). Interestingly, mouth-taping overnight, i.e., “forced” nasal breathing, improved the apnea-hypopnea index and reduced snoring in those with mild obstructive sleep apnea who mouth-breathed during sleep (103). Considering the acute benefits of nasal versus oral breathing in the present work, there is sufficient justification to examine whether mouth-taping overnight improves nighttime BP and/or BP throughout the subsequent day using automated BP monitoring. Such research could have important implications for this population if BP over several hours was reduced with nighttime nasal breathing as an intervention.

### Resting Heart Rate and HRV

We found that nasal compared with oral breathing did not affect heart rate or time-domain HRV metrics but did increase the LF contributions to HRV and decreased the LF/HF ratio. These changes to frequency-domain metrics of HRV suggest a greater parasympathetic to sympathetic dominance during nasal breathing (104–106). Our finding of breathing modulation (nasal vs. oral) changing HRV metrics has been observed in past work. One example is that device-guided slow breathing increases LF power for HRV (107–109). Consistent with this notion of altering autonomic nervous system balance, there are reports of device-guided slow breathing to reduce directly measured efferent muscle sympathetic outflow acutely (95, 96, 110) and chronically (111). However, other studies did not find the rate of breathing (from 7 to 21 breaths·min^−1^) to affect muscle sympathetic outflow acutely (112) or chronically (113, 114). In summary, nasal breathing may affect the autonomic nervous system as indicated by greater parasympathetic contributions to HRV but future studies with direct recordings of the nervous system (e.g., microneurography for muscle sympathetic outflow) are necessary to corroborate this.

### Resting BPV and cBRS

We found that one of six BPV metrics, systolic BP average real variability, was higher (i.e., worse) during nasal breathing. Although the effect size of the difference was very large, the 0.2-mmHg difference between conditions can be considered small (11). Therefore, we would contend that nasal versus oral breathing does not considerably affect beat-to-beat BP variability. Part of the rationale for measuring cBRS in the present study was a previous review concluding that increases in cBRS likely facilitate BP reductions during a single bout of breathing modulation (41). For example, slow breathing (e.g., 5–6 breaths·min^−1^) increased cBRS for up sequences only in one study (95) and all sequences in another (33). In opposition to our hypothesis, acute nasal compared with oral breathing did not affect cBRS in the subset of individuals with sufficient cBRS sequences during the resting periods. Future work should confirm these results for BPV and cBRS in a larger cohort of participants and determine whether there are differences between breathing routes in other populations.

### Resting RPE and RPB

We found that nasal compared with oral breathing was associated with significantly different RPE and RPB values at rest. The median RPE was 6 or “no exertion,” for both conditions. However, it is important to note that RPE was 6 for 19 of 20 participants (7, or “extremely light,” for the remaining individual) during nasal breathing and RPE was 6 for 13/20 participants (7 for six individuals and 8 for one individual) during oral breathing, resulting in a significantly greater proportion of adults reporting more than “no exertion” during oral breathing at rest. In addition, the median RPB was 0, or “nothing at all” during nasal breathing, and 0.5, or “very, very light (just noticeable)” during oral breathing. Thus, while the *P* values and effect sizes suggest a strong effect of the breathing route on perceived exertion and breathlessness, we would interpret the difference as very mild. That said, it is currently unclear whether this small difference would become more important over hours or days (e.g., preventing oral breathing via mouth-taping for a week) in healthy young adults. In patients with hyperventilation syndrome, nasal breathing reduces the severity of hyperventilation (115). So, between the present findings and the previous report from a patient population, there is justification for longer-term studies with the effect of nasal versus oral breathing on subjective ratings. Such future studies would benefit from complementary psychological assessments to provide additional insights.

### Exercising Data

We found that nasal compared with oral breathing did not affect BP, heart rate, or HRV metrics during submaximal exercise. One past study reported lower V̇o_2_ and breathing rates with nasal breathing during exercise at 60% of the maximum heart rate (39), suggestive of lower metabolic and respiratory demands. Thus, we reasoned that individuals could complete the same workload (e.g., 75 W) with a lower heart rate during nasal compared with oral breathing. However, our data do not support this idea. During maximal exercise, one (116), but not all (117), studies reported lower heart rate values with nasal versus oral breathing. However, these findings are difficult to interpret because participants completed an additional stage during the oral breathing trials. Second, these data were collected during maximal exercise whereas the present study aimed to examine the effects of breathing route on cardiovascular responses during submaximal exercise. To summarize, we did not find nasal breathing to affect several prognostic cardiovascular variables.

We also found that nasal breathing was associated with lower RPB, but not RPE, values during submaximal exercise. The median RPB values corresponded with “slight” breathlessness during nasal breathing and “moderate” breathlessness during oral breathing. We expected that nasal breathing would reduce RPE and RPB during exercise because the ventilatory response during exercise, which is attenuated with nasal versus oral breathing (39, 118), is linked to RPE and RPB (62, 64, 119–123). These variables are important because exercise-related breathlessness (i.e., exertional dyspnea) can reduce exercise participation (124, 125). One publication with relevance to this project concluded that oral breathing slightly increased RPE values relative to oronasal breathing during shuttle run testing in young males (126). Although our data did not reach statistical significance, our data (RPE of 13 vs. 12) are in agreement with the difference in RPE values (RPE of 19 vs. 18) in the past work, both having higher RPE values during oral breathing. To conclude, nasal breathing may have a modest effect on reducing RPB during acute submaximal exercise.

### Limitations

First, we considered the ecological validity and proof of concept nature of this research when deciding not to measure V̇o_2_, tidal volume, or end-tidal CO_2_ in this initial investigation on the acute cardiovascular effects of nasal breathing. This project was a necessary first step before deciding whether to interrogate potential mechanisms related to additional respiratory and/or psychological variables in future projects. Also, a breathing apparatus to collect and sample gases during nasal breathing could have confounded our findings and added undue difficulty to the present work. Nevertheless, we were able to directly address our primary and secondary hypotheses. Second, because nasal volume increases (127, 128) and nasal resistance decreases (129) after acute moderate-intensity cycling exercise for ∼20 min, it is possible that there was an order effect. However, the randomized design prevented this concern. Moreover, the reduction in resting diastolic BP from oral to nasal breathing did not differ between those who were randomized to oral breathing first and those who were randomized to nasal breathing first.

### Perspectives and Significance

At rest, nasal breathing was associated with lower BP and a greater parasympathetic contribution to HRV. During submaximal exercise, there were no differences between breathing routes on the cardiovascular variables assessed. These data suggest that nasal compared with oral breathing provides modest, but potentially clinically relevant, improvements in prognostic cardiovascular variables at rest, but not during exercise. This work advances our knowledge of how nasal breathing affects clinically relevant cardiovascular variables and provides foundational acute data in healthy young adults to justify future longer-term studies in other populations. Should these findings be confirmed in future studies, these data would provide partial support for a breathing modality with a high potential for translation (i.e., voluntarily breathing through the nose by keeping the lips sealed).

## DATA AVAILABILITY

Data are available upon reasonable request to the principal investigator after institutional data transfer approvals.

## GRANTS

This research was supported in part by the National Institutes of Health Grant K01HL160772 (to J.C.W.), American Heart Association Grant 23CDA1037938 (to J.C.W.), and an Undergraduate Research Opportunity Program Research Mentor Materials Grant (to J.C.W.).

## DISCLOSURES

No conflicts of interest, financial or otherwise, are declared by the authors.

## AUTHOR CONTRIBUTIONS

J.C.W., J.N.C., S.L.B., J.M., A.K.B., I.M.F., J.M.C., A.M.M., and K.F.K. conceived and designed research; J.C.W., J.N.C., S.L.B., J.M., A.K.B., I.M.F., J.M.C., A.M.M., and K.F.K. performed experiments; J.C.W., J.N.C., S.L.B., J.M., A.K.B., I.M.F., J.M.C., A.M.M., and K.F.K. analyzed data; J.C.W., J.N.C., S.L.B., J.M., A.K.B., I.M.F., J.M.C., A.M.M., and K.F.K. interpreted results of experiments; J.C.W. prepared figures; J.C.W. drafted manuscript; J.C.W., J.N.C., S.L.B., J.M., A.K.B., I.M.F., J.M.C., A.M.M., and K.F.K. edited and revised manuscript; J.C.W., J.N.C., S.L.B., J.M., A.K.B., I.M.F., J.M.C., A.M.M., and K.F.K. approved final version of manuscript.

## References

[1] Tsao CW, Aday AW, Almarzooq ZI, Anderson CAM, Arora P, Avery CL, , et al.; American Heart Association Council on Epidemiology and Prevention Statistics Committee and Stroke Statistics Subcommittee. Heart Disease and Stroke Statistics-2023 Update: a report from the American Heart Association. Circulation 147: e93–e621, 2023 [Erratum in *Circulation* 147: e622, 2023]. doi:10.1161/CIR.0000000000001123. 36695182 PMC12135016

[2] Hillebrand S, Gast KB, de Mutsert R, Swenne CA, Jukema JW, Middeldorp S, Rosendaal FR, Dekkers OM. Heart rate variability and first cardiovascular event in populations without known cardiovascular disease: meta-analysis and dose-response meta-regression. Europace 15: 742–749, 2013. doi:10.1093/europace/eus341. 23370966

[3] Fang SC, Wu YL, Tsai PS. Heart rate variability and risk of all-cause death and cardiovascular events in patients with cardiovascular disease: a meta-analysis of cohort studies. Biol Res Nurs 22: 45–56, 2020 [Erratum in *Biol Res Nurs* 22: 423–425, 2020]. doi:10.1177/1099800419877442. 31558032

[4] Asmuje NF, Mat S, Goh CH, Myint PK, Tan MP. Increased beat-to-beat blood pressure variability is associated with impaired cognitive function. Am J Hypertens 35: 998–1005, 2022. doi:10.1093/ajh/hpac107. 36153737

[5] Sible IJ, Jang JY, Dutt S, Yew B, Alitin JPM, Li Y, Blanken AE, Ho JK, Marshall AJ, Kapoor A, Shenasa F, Gaubert A, Nguyen A, Sturm VE, Mather M, Rodgers KE, Shao X, Wang DJ, Nation DA. Older adults with higher blood pressure variability exhibit cerebrovascular reactivity deficits. Am J Hypertens 36: 63–68, 2023. doi:10.1093/ajh/hpac108. 36149821 PMC9793985

[6] Filomena J, Riba-Llena I, Vinyoles E, Tovar JL, Mundet X, Castañé X, Vilar A, López-Rueda A, Jiménez-Baladó J, Cartanyà A, Montaner J, Delgado P, Investigators I; ISSYS Investigators. Short-term blood pressure variability relates to the presence of subclinical brain small vessel disease in primary hypertension. Hypertension 66: 634–640, 2015. doi:10.1161/HYPERTENSIONAHA.115.05440. 26101344

[7] Madden JM, O'Flynn AM, Dolan E, Fitzgerald AP, Kearney PM. Short-term blood pressure variability over 24 h and target organ damage in middle-aged men and women. J Hum Hypertens 29: 719–725, 2015. doi:10.1038/jhh.2015.18. 25787777

[8] Tatasciore A, Renda G, Zimarino M, Soccio M, Bilo G, Parati G, Schillaci G, De Caterina R. Awake systolic blood pressure variability correlates with target-organ damage in hypertensive subjects. Hypertension 50: 325–332, 2007. doi:10.1161/HYPERTENSIONAHA.107.090084. 17562971

[9] Mancia G, Bombelli M, Facchetti R, Madotto F, Corrao G, Trevano FQ, Grassi G, Sega R. Long-term prognostic value of blood pressure variability in the general population: results of the Pressioni Arteriose Monitorate e Loro Associazioni Study. Hypertension 49: 1265–1270, 2007. doi:10.1161/HYPERTENSIONAHA.107.088708. 17452502

[10] Pringle E, Phillips C, Thijs L, Davidson C, Staessen JA, de Leeuw PW, Jaaskivi M, Nachev C, Parati G, O'Brien ET, Tuomilehto J, Webster J, Bulpitt CJ, Fagard RH; Syst-Eur investigators. Systolic blood pressure variability as a risk factor for stroke and cardiovascular mortality in the elderly hypertensive population. J Hypertens 21: 2251–2257, 2003. doi:10.1097/00004872-200312000-00012. 14654744

[11] Wei FF, Li Y, Zhang L, Xu TY, Ding FH, Wang JG, Staessen JA. Beat-to-beat, reading-to-reading, and day-to-day blood pressure variability in relation to organ damage in untreated Chinese. Hypertension 63: 790–796, 2014. doi:10.1161/HYPERTENSIONAHA.113.02681. 24396027

[12] Billman GE, Schwartz PJ, Stone HL. Baroreceptor reflex control of heart rate: a predictor of sudden cardiac death. Circulation 66: 874–880, 1982. doi:10.1161/01.cir.66.4.874. 7116603

[13] Cerati D, Schwartz PJ. Single cardiac vagal fiber activity, acute myocardial ischemia, and risk for sudden death. Circ Res 69: 1389–1401, 1991. doi:10.1161/01.res.69.5.1389. 1934362

[14] La Rovere MT, Pinna GD, Raczak G. Baroreflex sensitivity: measurement and clinical implications. Ann Noninvasive Electrocardiol 13: 191–207, 2008. doi:10.1111/j.1542-474X.2008.00219.x. 18426445 PMC6931942

[15] Fisher JP, Zera T, Paton JFR. Respiratory-cardiovascular interactions. Handb Clin Neurol 188: 279–308, 2022. doi:10.1016/B978-0-323-91534-2.00006-0. 35965029

[16] Jalife J, Slenter V, Salata JJ, Michaels DC. Dynamic vagal control of pacemaker activity in the mammalian sinoatrial node. Circ Res 52: 642–656, 1983. doi:10.1161/01.res.52.6.642. 6861283

[17] Wang YP, Kuo TB, Lai CT, Chu JW, Yang CC. Effects of respiratory time ratio on heart rate variability and spontaneous baroreflex sensitivity. J Appl Physiol (1985) 115: 1648–1655, 2013. doi:10.1152/japplphysiol.00163.2013. 24092689

[18] Hautamäki H, Mikkola TS, Sovijärvi AR, Piirilä P, Haapalahti P. Menopausal hot flushes do not associate with changes in heart rate variability in controlled testing: a randomized trial on hormone therapy. Acta Obstet Gynecol Scand 92: 902–908, 2013. doi:10.1111/aogs.12164. 23656530

[19] Madden K, Savard GK. Effects of mental state on heart rate and blood pressure variability in men and women. Clin Physiol 15: 557–569, 1995. doi:10.1111/j.1475-097x.1995.tb00544.x. 8590551

[20] Pober DM, Braun B, Freedson PS. Effects of a single bout of exercise on resting heart rate variability. Med Sci Sports Exerc 36: 1140–1148, 2004. doi:10.1249/01.mss.0000132273.30827.9a. 15235317

[21] Chapman CL, Reed EL, Worley ML, Pietrafesa LD, Kueck PJ, Bloomfield AC, Schlader ZJ, Johnson BD. Sugar-sweetened soft drink consumption acutely decreases spontaneous baroreflex sensitivity and heart rate variability. Am J Physiol Regul Integr Comp Physiol 320: R641–R652, 2021. doi:10.1152/ajpregu.00310.2020. 33533320 PMC8163604

[22] Babcock MC, DuBose LE, Hildreth KL, Stauffer BL, Cornwell WK 3rd, Kohrt WM, Moreau KL. Age-associated reductions in cardiovagal baroreflex sensitivity are exaggerated in middle-aged and older men with low testosterone. J Appl Physiol 133: 403–415, 2022. doi:10.1152/japplphysiol.00245.2022. 35771224 PMC9359637

[23] Fisher JP, Roche J, Turner R, Walzl A, Roveri G, Gatterer H, Siebenmann C. Hypobaric hypoxia and cardiac baroreflex sensitivity in young women. Am J Physiol Heart Circ Physiol 323: H1048–H1054, 2022. doi:10.1152/ajpheart.00452.2022. 36240437 PMC9678423

[24] Vierra J, Boonla O, Prasertsri P. Effects of sleep deprivation and 4-7-8 breathing control on heart rate variability, blood pressure, blood glucose, and endothelial function in healthy young adults. Physiol Rep 10: e15389, 2022. doi:10.14814/phy2.15389. 35822447 PMC9277512

[25] Katona PG, Jih F. Respiratory sinus arrhythmia: noninvasive measure of parasympathetic cardiac control. J Appl Physiol 39: 801–805, 1975. doi:10.1152/jappl.1975.39.5.801. 1184518

[26] DeBeck LD, Petersen SR, Jones KE, Stickland MK. Heart rate variability and muscle sympathetic nerve activity response to acute stress: the effect of breathing. Am J Physiol Regul Integr Comp Physiol 299: R80–R91, 2010. doi:10.1152/ajpregu.00246.2009. 20410469 PMC5017870

[27] Brown TE, Beightol LA, Koh J, Eckberg DL. Important influence of respiration on human RR interval power spectra is largely ignored. J Appl Physiol (1985) 75: 2310–2317, 1993. doi:10.1152/jappl.1993.75.5.2310. 8307890

[28] Perini R, Veicsteinas A. Heart rate variability and autonomic activity at rest and during exercise in various physiological conditions. Eur J Appl Physiol 90: 317–325, 2003. doi:10.1007/s00421-003-0953-9. 13680241

[29] Fontolliet T, Gianella P, Pichot V, Barthélémy JC, Gasche-Soccal P, Ferretti G, Lador F. Heart rate variability and baroreflex sensitivity in bilateral lung transplant recipients. Clin Physiol Funct Imaging 38: 872–880, 2018. doi:10.1111/cpf.12499. 29316181

[30] Busha BF, Hage E, Hofmann C. Gender and breathing route modulate cardio-respiratory variability in humans. Respir Physiol Neurobiol 166: 87–94, 2009. doi:10.1016/j.resp.2009.02.008. 19429524

[31] Bernardi L, Porta C, Gabutti A, Spicuzza L, Sleight P. Modulatory effects of respiration. Auton Neurosci 90: 47–56, 2001. doi:10.1016/S1566-0702(01)00267-3. 11485292

[32] Joseph CN, Porta C, Casucci G, Casiraghi N, Maffeis M, Rossi M, Bernardi L. Slow breathing improves arterial baroreflex sensitivity and decreases blood pressure in essential hypertension. Hypertension 46: 714–718, 2005. doi:10.1161/01.HYP.0000179581.68566.7d. 16129818

[33] Radaelli A, Raco R, Perfetti P, Viola A, Azzellino A, Signorini MG, Ferrari AU. Effects of slow, controlled breathing on baroreceptor control of heart rate and blood pressure in healthy men. J Hypertens 22: 1361–1370, 2004. doi:10.1097/01.hjh.0000125446.28861.51. 15201553

[34] Rodrigues GD, Nobrega A, Soares P. Respiratory training in older women: unravelling central and peripheral hemodynamic slow oscillatory patterns. Exp Gerontol 172: 112058, 2023. doi:10.1016/j.exger.2022.112058. 36529363

[35] Walker A, Surda P, Rossiter M, Little S. Nasal function and dysfunction in exercise. J Laryngol Otol 130: 431–434, 2016. doi:10.1017/S0022215116000128. 27095550

[36] Tomori Z, Widdicombe JG. Muscular, bronchomotor and cardiovascular reflexes elicited by mechanical stimulation of the respiratory tract. J Physiol 200: 25–49, 1969. doi:10.1113/jphysiol.1969.sp008680. 5761951 PMC1350416

[37] Bizjak DA, Schams P, Bloch W, Grau M, Latsch J. The intranasal AlaxoLito Plus Nasal Stent: improvement of NO-induced microrheology and oxygen uptake during exercise? Respir Physiol Neurobiol 269: 103260, 2019. doi:10.1016/j.resp.2019.103260. 31352012

[38] Trevisan ME, Boufleur J, Soares JC, Haygert CJ, Ries LG, Corrêa EC. Diaphragmatic amplitude and accessory inspiratory muscle activity in nasal and mouth-breathing adults: a cross-sectional study. J Electromyogr Kinesiol 25: 463–468, 2015. doi:10.1016/j.jelekin.2015.03.006. 25900327

[39] Hall RL. Energetics of nose and mouth breathing, body size, body composition, and nose volume in young adult males and females. Am J Hum Biol 17: 321–330, 2005. doi:10.1002/ajhb.20122. 15849711

[40] Cipriano GF, Cipriano G Jr, Santos FV, Güntzel Chiappa AM, Pires L, Cahalin LP, Chiappa GR. Current insights of inspiratory muscle training on the cardiovascular system: a systematic review with meta-analysis. Integr Blood Press Control 12: 1–11, 2019. doi:10.2147/IBPC.S159386. 31190975 PMC6535083

[41] Pingali H, Hunter SD. Exploring mechanisms of blood pressure regulation in response to device-guided and non-device-guided slow breathing: a mini review. Auton Neurosci 244: 103050, 2023. doi:10.1016/j.autneu.2022.103050. 36410208

[42] Mengden T, Bachler M, Sehnert W, Marschall P, Wassertheurer S. Device-guided slow breathing with direct biofeedback of pulse wave velocity—acute effects on pulse arrival time and self-measured blood pressure. Blood Press Monit 28: 52–58, 2023 [Erratum in *Blood Press Monit* 28: 121, 2023]. doi:10.1097/MBP.0000000000000628. 36606480 PMC9815813

[43] Bachler M, Sehnert W, Mikisek I, Wassertheurer S, Mengden T. Non-invasive quantification of the effect of device-guided slow breathing with direct feedback to the patient to reduce blood pressure. Physiol Meas 41: 104002, 2020. doi:10.1088/1361-6579/abb320. 33164912

[44] Lin IM. Effects of a cardiorespiratory synchronization training mobile application on heart rate variability and electroencephalography in healthy adults. Int J Psychophysiol 134: 168–177, 2018. doi:10.1016/j.ijpsycho.2018.09.005. 30243751

[45] Tavoian D, Craighead DH. Deep breathing exercise at work: potential applications and impact. Front Physiol 14: 1040091, 2023. doi:10.3389/fphys.2023.1040091. 36711016 PMC9877284

[46] Balban MY, Neri E, Kogon MM, Weed L, Nouriani B, Jo B, Holl G, Zeitzer JM, Spiegel D, Huberman AD. Brief structured respiration practices enhance mood and reduce physiological arousal. Cell Rep Med 4: 100895, 2023. doi:10.1016/j.xcrm.2022.100895. 36630953 PMC9873947

[47] Conway J, Boon N, Davies C, Jones JV, Sleight P. Neural and humoral mechanisms involved in blood pressure variability. J Hypertens 2: 203–208, 1984. doi:10.1097/00004872-198404000-00013. 6398335

[48] Yasuda Y, Itoh T, Miyamura M, Nishino H. Comparison of exhaled nitric oxide and cardiorespiratory indices between nasal and oral breathing during submaximal exercise in humans. Jpn J Physiol 47: 465–470, 1997. doi:10.2170/jjphysiol.47.465. 9504133

[49] LaComb CO, Tandy RD, Lee SP, Young JC, Navalta JW. Oral versus nasal breathing during moderate to high intensity submaximal aerobic exercise. Int J Kinesiol Sports Sci 5: 8–16, 2017. doi:10.7575//aiac.ijkss.v.5n.1p.8.

[50] Tzemos N, Lim PO, Mackenzie IS, MacDonald TM. Exaggerated exercise blood pressure response and future cardiovascular disease. J Clin Hypertens (Greenwich) 17: 837–844, 2015. doi:10.1111/jch.12629. 26235814 PMC8032021

[51] Miyai N, Arita M, Miyashita K, Morioka I, Shiraishi T, Nishio I. Blood pressure response to heart rate during exercise test and risk of future hypertension. Hypertension 39: 761–766, 2002. doi:10.1161/hy0302.105777. 11897759

[52] Moore MN, Climie RE, Otahal P, Schultz MG. Exercise blood pressure and cardiovascular disease risk: a systematic review and meta-analysis of cross-sectional studies. J Hypertens 39: 2395–2402, 2021. doi:10.1097/HJH.0000000000002962. 34738988

[53] Schultz MG, La Gerche A, Sharman JE. Blood pressure response to exercise and cardiovascular disease. Curr Hypertens Rep 19: 89, 2017. doi:10.1007/s11906-017-0787-1. 29046978

[54] Recinto C, Efthemeou T, Boffelli PT, Navalta JW. Effects of nasal or oral breathing on anaerobic power output and metabolic responses. Int J Exerc Sci 10: 506–514, 2017. 28674596 10.70252/EHDR7442 PMC5466403

[55] Craig CL, Marshall AL, Sjöström M, Bauman AE, Booth ML, Ainsworth BE, Pratt M, Ekelund U, Yngve A, Sallis JF, Oja P. International physical activity questionnaire: 12-country reliability and validity. Med Sci Sports Exerc 35: 1381–1395, 2003. doi:10.1249/01.MSS.0000078924.61453.FB. 12900694

[56] Borg GA. Psychophysical bases of perceived exertion. Med Sci Sports Exerc 14: 377–381, 1982. 7154893

[57] Dyspnea. Mechanisms, assessment, and management: a consensus statement. American Thoracic Society. Am J Respir Crit Care Med 159: 321–340, 1999. doi:10.1164/ajrccm.159.1.ats898. 9872857

[58] Ainsworth BE, Haskell WL, Herrmann SD, Meckes N, Bassett DR Jr, Tudor-Locke C, Greer JL, Vezina J, Whitt-Glover MC, Leon AS. 2011 Compendium of Physical Activities: a second update of codes and MET values. Med Sci Sports Exerc 43: 1575–1581, 2011. doi:10.1249/MSS.0b013e31821ece12. 21681120

[59] Niinimaa V, Cole P, Mintz S, Shephard RJ. The switching point from nasal to oronasal breathing. Respir Physiol 42: 61–71, 1980. doi:10.1016/0034-5687(80)90104-8. 7444224

[60] Perrin C, Lacoste J, Mari R. A nasal functional test: the opening of mouth during physical effort. Rhinology 15: 33–38, 1977. 918511

[61] James DS, Lambert WE, Mermier CM, Stidley CA, Chick TW, Samet JM. Oronasal distribution of ventilation at different ages. Arch Environ Health 52: 118–123, 1997. doi:10.1080/00039899709602874. 9124871

[62] Babb TG, Ranasinghe KG, Comeau LA, Semon TL, Schwartz B. Dyspnea on exertion in obese women: association with an increased oxygen cost of breathing. Am J Respir Crit Care Med 178: 116–123, 2008. doi:10.1164/rccm.200706-875OC. 18420968

[63] Bhammar DM, Babb TG, Xu ML, Bates JHT. Impact of insulin resistance on asthma: is there truly no role of “obesity”? Am J Respir Crit Care Med 207: 110–111, 2023. doi:10.1164/rccm.202209-1828LE. 36260829 PMC9952868

[64] Bernhardt V, Wood HE, Moran RB, Babb TG. Dyspnea on exertion in obese men. Respir Physiol Neurobiol 185: 241–248, 2013. doi:10.1016/j.resp.2012.10.007. 23085240 PMC3529969

[65] Babcock MC, Robinson AT, Watso JC, Migdal KU, Martens CR, Edwards DG, Pescatello LS, Farquhar WB. Salt loading blunts central and peripheral postexercise hypotension. Med Sci Sports Exerc 52: 935–943, 2020. doi:10.1249/MSS.0000000000002187. 31609296 PMC7144834

[66] Babcock MC, Brian MS, Watso JC, Edwards DG, Stocker SD, Wenner MM, Farquhar WB. Alterations in dietary sodium intake affect cardiovagal baroreflex sensitivity. Am J Physiol Regul Integr Comp Physiol 315: R688–R695, 2018. doi:10.1152/ajpregu.00002.2018. 29949407 PMC6230891

[67] Linder BA, Babcock MC, Pollin KU, Watso JC, Robinson AT. Short-term high salt intake does not influence resting or exercising heart rate variability but increases MCP-1 concentration in healthy young adults. Am J Physiol Regul Integr Comp Physiol 324: R666–R676, 2023. doi:10.1152/ajpregu.00240.2022. 36939211 PMC10110701

[68] Staley R, Garcia RG, Stowell J, Sclocco R, Fisher H, Napadow V, Goldstein JM, Barbieri R. Modulatory effects of respiratory-gated auricular vagal nerve stimulation on cardiovagal activity in hypertension. Annu Int Conf IEEE Eng Med Biol Soc 2020:2581–2584, 2020. doi:10.1109/EMBC44109.2020.9175768. 33018534

[69] Shaffer F, Ginsberg JP. An overview of heart rate variability metrics and norms. Front Public Health 5: 258, 2017. doi:10.3389/fpubh.2017.00258. 29034226 PMC5624990

[70] Michael S, Graham KS, Davis GMO. Cardiac autonomic responses during exercise and post-exercise recovery using heart rate variability and systolic time intervals—a review. Front Physiol 8: 301, 2017. doi:10.3389/fphys.2017.00301. 28611675 PMC5447093

[71] Guelen I, Westerhof BE, Van Der Sar GL, Van Montfrans GA, Kiemeneij F, Wesseling KH, Bos WJ. Finometer, finger pressure measurements with the possibility to reconstruct brachial pressure. Blood Press Monit 8: 27–30, 2003. doi:10.1097/00126097-200302000-00006. 12604933

[72] Watso JC, Babcock MC, Robinson AT, Migdal KU, Wenner MM, Stocker SD, Farquhar WB. Water deprivation does not augment sympathetic or pressor responses to sciatic afferent nerve stimulation in rats or to static exercise in humans. J Appl Physiol (1985) 127: 235–245, 2019. doi:10.1152/japplphysiol.00005.2019. 31070954 PMC6692747

[73] Watso JC, Robinson AT, Babcock MC, Migdal KU, Wenner MM, Stocker SD, Farquhar WB. Short-term water deprivation does not increase blood pressure variability or impair neurovascular function in healthy young adults. Am J Physiol Regul Integr Comp Physiol 318: R112–R121, 2019. doi:10.1152/ajpregu.00149.2019. 31617739 PMC6985800

[74] Watso JC, Robinson AT, Babcock MC, Migdal KU, Witman MAH, Wenner MM, Stocker SD, Farquhar WB. Short-term water deprivation attenuates the exercise pressor reflex in older female adults. Physiol Rep 8: e14581, 2020. doi:10.14814/phy2.14581. 32965797 PMC7510566

[75] Watso JC, Huang M, Moralez G, Cramer MN, Hendrix JM, Cimino FA 3rd, Belval LN, Hinojosa-Laborde C, Crandall CG. Low dose ketamine reduces pain perception and blood pressure, but not muscle sympathetic nerve activity, responses during a cold pressor test. J Physiol 599: 67–81, 2021. doi:10.1113/JP280706. 33017047

[76] Watso JC, Babcock MC, Migdal KU, Farquhar WB, Robinson AT. The relation between habitual physical activity and sympathetic vascular transduction in healthy young adults. Clin Auton Res 31: 335–337, 2021. doi:10.1007/s10286-021-00770-0. 33475888 PMC8043983

[77] Watso JC, Huang M, Belval LN, Cimino FA, Jarrard CP, Hendrix JM, Hinojosa-Laborde C, Crandall CG. Low-dose fentanyl reduces pain perception, muscle sympathetic nerve activity responses, and blood pressure responses during the cold pressor test. Am J Physiol Regul Integr Comp Physiol 322: R64–R76, 2022 [Erratum in *Am J Physiol Regul Integr Comp Physiol* 323: R277, 2022]. doi:10.1152/ajpregu.00218.2021. 34851729 PMC8742733

[78] Watso JC, Belval LN, Cimino FA 3rd, Orth BD, Hendrix JM, Huang M, Johnson E, Foster J, Hinojosa-Laborde C, Crandall CG. Low-dose morphine reduces tolerance to central hypovolemia in healthy adults without affecting muscle sympathetic outflow. Am J Physiol Heart Circ Physiol 323: H89–H99, 2022. doi:10.1152/ajpheart.00091.2022. 35452317 PMC9190738

[79] Watso JC, Belval LN, Cimino FA 3rd, Orth BD, Hendrix JM, Huang M, Johnson E, Foster J, Hinojosa-Laborde C, Crandall CG. Low-dose morphine reduces pain perception and blood pressure, but not muscle sympathetic outflow, responses during the cold pressor test. Am J Physiol Heart Circ Physiol 323: H223–H234, 2022. doi:10.1152/ajpheart.00092.2022. 35714174 PMC9273278

[80] Wesseling KH, Jansen JR, Settels JJ, Schreuder JJ. Computation of aortic flow from pressure in humans using a nonlinear, three-element model. J Appl Physiol (1985) 74: 2566–2573, 1993. doi:10.1152/jappl.1993.74.5.2566. 8335593

[81] Jansen JR, Schreuder JJ, Mulier JP, Smith NT, Settels JJ, Wesseling KH. A comparison of cardiac output derived from the arterial pressure wave against thermodilution in cardiac surgery patients. Br J Anaesth 87: 212–222, 2001. doi:10.1093/bja/87.2.212. 11493492

[82] Berger A, Grossman E, Katz M, Kivity S, Klempfner R, Segev S, Goldenberg I, Sidi Y, Maor E. Exercise systolic blood pressure variability is associated with increased risk for new-onset hypertension among normotensive adults. J Am Soc Hypertens 10: 527–535.e2, 2016. doi:10.1016/j.jash.2016.04.003. 27292824

[83] Boggia J, Asayama K, Li Y, Hansen TW, Mena L, Schutte R. Cardiovascular risk stratification and blood pressure variability on ambulatory and home blood pressure measurement. Curr Hypertens Rep 16: 470, 2014. doi:10.1007/s11906-014-0470-8. 25097109

[84] Mena LJ, Felix VG, Melgarejo JD, Maestre GE. 24-Hour blood pressure variability assessed by average real variability: a systematic review and meta-analysis. J Am Heart Assoc 6: 1–10, 2017. doi:10.1161/JAHA.117.006895. 29051214 PMC5721878

[85] Bertinieri G, di Rienzo M, Cavallazzi A, Ferrari AU, Pedotti A, Mancia G. A new approach to analysis of the arterial baroreflex. J Hypertens Suppl 3: S79–S81, 1985. 2856787

[86] Gandevia S. Publications, replication and statistics in physiology plus two neglected curves. J Physiol 599: 1719–1721, 2021. doi:10.1113/JP281360. 33507571

[87] Curran-Everett D. Evolution in statistics: *P* values, statistical significance, kayaks, and walking trees. Adv Physiol Educ 44: 221–224, 2020. doi:10.1152/advan.00054.2020. 32412384

[88] Lakens D. Calculating and reporting effect sizes to facilitate cumulative science: a practical primer for *t*-tests and ANOVAs. Front Psychol 4: 863, 2013. doi:10.3389/fpsyg.2013.00863. 24324449 PMC3840331

[89] Kerby D. The simple difference formula: an approach to teaching nonparametric correlation. Compr Psychol 3: 1–9, 2014. doi:10.2466/11.IT.3.1.

[90] The jamovi project. jamovi (Version 2.2) [Computer Software]. 2022 [Accessed 9 May 2023]. https://www.jamovi.org.

[91] Saito I, Takata Y, Maruyama K, Eguchi E, Kato T, Shirahama R, Tomooka K, Kawamura R, Sano M, Tabara Y, Osawa H, Tanigawa T. Association between heart rate variability and home blood pressure: the Toon Health Study. Am J Hypertens 31: 1120–1126, 2018. doi:10.1093/ajh/hpy100. 29982275

[92] Bolin LP, Saul AD, Bethune Scroggs LL, Horne C. A pilot study investigating the relationship between heart rate variability and blood pressure in young adults at risk for cardiovascular disease. Clin Hypertens 28: 2, 2022. doi:10.1186/s40885-021-00185-z. 35031077 PMC8760819

[93] Ferris BG Jr, Mead J, Opie LH. Partitioning of respiratory flow resistance in man. J Appl Physiol 19: 653–658, 1964. doi:10.1152/jappl.1964.19.4.653. 14195575

[94] Nuckowska MK, Gruszecki M, Kot J, Wolf J, Guminski W, Frydrychowski AF, Wtorek J, Narkiewicz K, Winklewski PJ. Impact of slow breathing on the blood pressure and subarachnoid space width oscillations in humans. Sci Rep 9: 6232, 2019. doi:10.1038/s41598-019-42552-9. 30996273 PMC6470142

[95] Adler TE, Coovadia Y, Cirone D, Khemakhem ML, Usselman CW. Device-guided slow breathing reduces blood pressure and sympathetic activity in young normotensive individuals of both sexes. J Appl Physiol (1985) 127: 1042–1049, 2019. doi:10.1152/japplphysiol.00442.2019. 31436511

[96] Fonkoue IT, Marvar PJ, Norrholm SD, Kankam ML, Li Y, DaCosta D, Rothbaum BO, Park J. Acute effects of device-guided slow breathing on sympathetic nerve activity and baroreflex sensitivity in posttraumatic stress disorder. Am J Physiol Heart Circ Physiol 315: H141–H149, 2018. doi:10.1152/ajpheart.00098.2018. 29652544 PMC6087774

[97] Zhang Z, Wang B, Wu H, Chai X, Wang W, Peng C-K. Effects of slow and regular breathing exercise on cardiopulmonary coupling and blood pressure. Med Biol Eng Comput 55: 327–341, 2017. doi:10.1007/s11517-016-1517-6. 27193228

[98] Grossman E, Grossman A, Schein MH, Zimlichman R, Gavish B. Breathing-control lowers blood pressure. J Hum Hypertens 15: 263–269, 2001. doi:10.1038/sj.jhh.1001147. 11319675

[99] Schein MH, Gavish B, Herz M, Rosner-Kahana D, Naveh P, Knishkowy B, Zlotnikov E, Ben-Zvi N, Melmed RN. Treating hypertension with a device that slows and regularises breathing: a randomised, double-blind controlled study. J Hum Hypertens 15: 271–278, 2001. doi:10.1038/sj.jhh.1001148. 11319676

[100] Rosenthal T, Alter A, Peleg E, Gavish B. Device-guided breathing exercises reduce blood pressure: ambulatory and home measurements. Am J Hypertens 14: 74–76, 2001. doi:10.1016/s0895-7061(00)01235-8. 11206685

[101] Viskoper R, Shapira I, Priluck R, Mindlin R, Chornia L, Laszt A, Dicker D, Gavish B, Alter A. Nonpharmacologic treatment of resistant hypertensives by device-guided slow breathing exercises. Am J Hypertens 16: 484–487, 2003. doi:10.1016/s0895-7061(03)00571-5. 12799098

[102] Craighead DH, Tavoian D, Freeberg KA, Mazzone JL, Vranish JR, DeLucia CM, Seals DR, Bailey EF. A multi-trial, retrospective analysis of the antihypertensive effects of high-resistance, low-volume inspiratory muscle strength training. J Appl Physiol 133: 1001–1010, 2022. doi:10.1152/japplphysiol.00425.2022. 36107991 PMC9550580

[103] Lee YC, Lu CT, Cheng WN, Li HY. The impact of mouth-taping in mouth-breathers with mild obstructive sleep apnea: a preliminary study. Healthcare (Basel) 10: 1755, 2022. doi:10.3390/healthcare10091755. 36141367 PMC9498537

[104] Singh N, Moneghetti KJ, Christle JW, Hadley D, Plews D, Froelicher V. Heart rate variability: an old metric with new meaning in the era of using mhealth technologies for health and exercise training guidance. Part one: physiology and methods. Arrhythm Electrophysiol Rev 7: 193–198, 2018. doi:10.15420/aer.2018.27.2. 30416733 PMC6141929

[105] Heart rate variability: standards of measurement, physiological interpretation, and clinical use. Task Force of the European Society of Cardiology and the North American Society of Pacing and Electrophysiology. Eur Heart J 17: 354–381, 1996. 8737210

[106] Valentini M, Parati G. Variables influencing heart rate. Prog Cardiovasc Dis 52: 11–19, 2009. doi:10.1016/j.pcad.2009.05.004. 19615488

[107] Howorka K, Pumprla J, Tamm J, Schabmann A, Klomfar S, Kostineak E, Howorka N, Sovova E. Effects of guided breathing on blood pressure and heart rate variability in hypertensive diabetic patients. Auton Neurosci 179: 131–137, 2013. doi:10.1016/j.autneu.2013.08.065. 24021938

[108] Wang CH, Yang HW, Huang HL, Hsiao CY, Jiu BK, Lin C, Lo MT. Long-term effect of device-guided slow breathing on blood pressure regulation and chronic inflammation in patients with essential hypertension using a wearable ECG device. Acta Cardiol Sin 37: 195–203, 2021. doi:10.6515/ACS.202103_37(2).20200907A. 33716462 PMC7953112

[109] Anderson DE, McNeely JD, Windham BG. Device-guided slow-breathing effects on end-tidal CO(2) and heart-rate variability. Psychol Health Med 14: 667–679, 2009. doi:10.1080/13548500903322791. 20183539 PMC4054864

[110] Hering D, Kucharska W, Kara T, Somers VK, Parati G, Narkiewicz K. Effects of acute and long-term slow breathing exercise on muscle sympathetic nerve activity in untreated male patients with hypertension. J Hypertens 31: 739–746, 2013. doi:10.1097/HJH.0b013e32835eb2cf. 23385649

[111] Oneda B, Ortega KC, Gusmão JL, Araújo TG, Mion D Jr. Sympathetic nerve activity is decreased during device-guided slow breathing. Hypertens Res 33: 708–712, 2010. doi:10.1038/hr.2010.74. 20520613

[112] Limberg JK, Morgan BJ, Schrage WG, Dempsey JA. Respiratory influences on muscle sympathetic nerve activity and vascular conductance in the steady state. Am J Physiol Heart Circ Physiol 304: H1615–H1623, 2013. doi:10.1152/ajpheart.00112.2013. 23585141 PMC3680774

[113] Fonkoue IT, Hu Y, Jones T, Vemulapalli M, Sprick JD, Rothbaum B, Park J. Eight weeks of device-guided slow breathing decreases sympathetic nervous reactivity to stress in posttraumatic stress disorder. Am J Physiol Regul Integr Comp Physiol 319: R466–R475, 2020. doi:10.1152/ajpregu.00079.2020. 32847397 PMC7642907

[114] de Barros S, da Silva GV, de Gusmão JL, de Araújo TG, de Souza DR, Cardoso CG Jr, Oneda B, Mion D Jr. Effects of long term device-guided slow breathing on sympathetic nervous activity in hypertensive patients: a randomized open-label clinical trial. Blood Press 26: 359–365, 2017. doi:10.1080/08037051.2017.1357109. 28724309

[115] Gouzi F, Dubois-Gamez AS, Lacoude P, Abdellaoui A, Hédon C, Charriot J, Boissin C, Vachier I, Hayot M, Molinari N, Bourdin A. Feasibility of a nasal breathing training during pulmonary rehabilitation. A pilot randomized controlled study. Respir Physiol Neurobiol 308: 103987, 2023. doi:10.1016/j.resp.2022.103987. 36372120

[116] Chinevere TD, Faria EW, Faria IE. Nasal splinting effects on breathing patterns and cardiorespiratory responses. J Sports Sci 17: 443–447, 1999. doi:10.1080/026404199365759. 10404493

[117] Morton AR, King K, Papalia S, Goodman C, Turley KR, Wilmore JH. Comparison of maximal oxygen consumption with oral and nasal breathing. Aust J Sci Med Sport 27: 51–55, 1995. 8599744

[118] Harbour E, Stöggl T, Schwameder H, Finkenzeller T. Breath tools: a synthesis of evidence-based breathing strategies to enhance human running. Front Physiol 13: 813243, 2022. doi:10.3389/fphys.2022.813243. 35370762 PMC8967998

[119] Bernhardt V, Babb TG. Respiratory symptom perception differs in obese women with strong or mild breathlessness during constant-load exercise. Chest 145: 361–369, 2014. doi:10.1378/chest.12-2885. 23989732 PMC3913302

[120] Ofir D, Laveneziana P, Webb KA, O'Donnell DE. Ventilatory and perceptual responses to cycle exercise in obese women. J Appl Physiol (1985) 102: 2217–2226, 2007. doi:10.1152/japplphysiol.00898.2006. 17234804

[121] Romagnoli I, Laveneziana P, Clini EM, Palange P, Valli G, De Blasio F, Gigliotti F, Scano G. Role of hyperinflation vs. deflation on dyspnoea in severely to extremely obese subjects. Acta Physiol (Oxf) 193: 393–402, 2008. doi:10.1111/j.1748-1716.2008.01852.x. 18363899

[122] Sin DD, Jones RL, Man SF. Obesity is a risk factor for dyspnea but not for airflow obstruction. Arch Intern Med 162: 1477–1481, 2002. doi:10.1001/archinte.162.13.1477. 12090884

[123] Whipp BJ, Davis JA. The ventilatory stress of exercise in obesity. Am Rev Respir Dis 129: S90–S92, 1984. doi:10.1164/arrd.1984.129.2P2.S90. 6696351

[124] Balmain BN, Weinstein K, Bernhardt V, Marines-Price R, Tomlinson AR, Babb TG. Multidimensional aspects of dyspnea in obese patients referred for cardiopulmonary exercise testing. Respir Physiol Neurobiol 274: 103365, 2020. doi:10.1016/j.resp.2019.103365. 31899350 PMC7002243

[125] Chlif M, Chaouachi A, Ahmaidi S. Effect of aerobic exercise training on ventilatory efficiency and respiratory drive in obese subjects. Respir Care 62: 936–946, 2017. doi:10.4187/respcare.04923. 28442632

[126] Meir R, Zhao GG, Zhou S, Beavers R, Davie A. The acute effect of mouth only breathing on time to completion, heart rate, rate of perceived exertion, blood lactate, and ventilatory measures during a high-intensity shuttle run sequence. J Strength Cond Res 28: 950–957, 2014. doi:10.1519/JSC.0000000000000246. 24077371

[127] Fonseca MT, Machado JA, Pereira SA, Pinto KM, Voegels RL. Effects of physical exercise in nasal volume. Braz J Otorhinolaryngol 72: 256–260, 2006. doi:10.1016/s1808-8694(15)30065-3. 16951862 PMC9445755

[128] Fonseca MT, Voegels RL, Pinto KM. Evaluation of nasal volume by acoustic rhinometry before and after physical exercise. Am J Rhinol 20: 269–273, 2006. doi:10.2500/ajr.2006.20.2863. 16871927

[129] Strohl KP, Decker MJ, Olson LG, Flak TA, Hoekje PL. The nasal response to exercise and exercise induced bronchoconstriction in normal and asthmatic subjects. Thorax 43: 890–895, 1988. doi:10.1136/thx.43.11.890. 3222760 PMC461549

